# Less water, more seeds? The E3 ligase TaGW2 regulates drought resistance in wheat

**DOI:** 10.1093/plcell/koad311

**Published:** 2023-12-18

**Authors:** Humberto Herrera-Ubaldo

**Affiliations:** Assistant Features Editor, The Plant Cell, American Society of Plant Biologists; Department of Plant Sciences, University of Cambridge, Cambridge CB2 3EA, UK

Water deficit is a limiting factor for agriculture and can severely affect crop yield (Eckardt et al. 2023). Physiological responses to drought commonly include the increase of water uptake, control of water loss, and osmotic adjustment within tissues. These processes are tightly controlled at the gene expression level and commonly involve abscisic acid, brassinosteroids, and other hormone signaling pathways (Gupta et al. 2020). Current crop breeding programs are focused on enhancing plant resistance to harsh environments while maintaining or increasing yield. In the effort to increase drought resistance or yield in wheat, some genes have been identified. For instance, *TaGW2*, which controls grain size in wheat (Zhang et al. 2018), increases plant survival after drought stress when overexpressed (Li et al. 2015); however, it also causes a reduction in yield. Thus, finding a good balance between drought resistance and yield is critical for breeding programs.

In this issue, **Shumin Li**, **Yifang Zhang, Yuling Liu, and colleagues** (Li et al. 2023) dissected the signaling downstream of TaGW2 and identified TaARR12 as its target and regulator of drought response. They describe how a fine-tuning regulation of the TaGW2-TaARR12 module can increase drought resistance and yield in wheat.

Lines overexpressing *TaGW2* displayed reduced kernel size and weight and good recovery after drought. On the other hand, *TaGW2* knockdown (RNAi) lines increased in kernel size and weight, but recovery after drought was compromised. Analysis of the knockout mutant *tagw2* (Zhang et al. 2018) confirmed this phenotype. The lines displayed differences in water loss rates, stomatal apertures, and water use efficiency (photosynthetic rate relative to transpiration). The expression of *TaGW2* increases during drought stress or treatments with abscisic acid, suggesting a key role in activating stress responses. This function was confirmed with gene expression analysis on *tagw2* and KN199 (wt), which revealed the downregulation of stress-responsive genes related to several physiological processes.

The role of GW2 in controlling grain size was first characterized in rice (Song et al. 2007). This protein is an E3 class ubiquitin ligase, which was reported to guide the degradation of WIDE GRAIN 1 (Hao et al. 2021). The ubiquitination-mediated protein degradation involves 3 enzymes: E1, which performs the ubiquitin (Ub) activation; E2, which catalyzes Ub-conjugation; and E3, the Ub-ligase. To identify degradation targets of TaGW2 in wheat, the authors first conducted ubiquitylome profiling of the *tagw2* and KN199 lines and identified 159 proteins with increased ubiquitination levels and 403 proteins with decreased levels. They identified 83 proteins with increased abundance in the proteome of *tagw2* that also presented a decrease in Ub levels, suggesting they are targets of TaGW2 ubiquitination. Another way to identify ubiquitination targets is by detecting physical interaction. So, the authors conducted a yeast 2-hybrid assay with TaGW2 as a bait. The 68 protein interactions detected were cross-checked with the ubiquitylome and proteome data. They found 9 candidates with decreased ubiquitination levels and increased abundance in *tagw2*, strongly suggesting that they are targets of TaGW2 for ubiquitination. One of those proteins was TaARR12, a type-B ARR transcription factor in the cytokinin signaling pathway. Further experiments confirmed that TaGW2 and TaARR12 physically interact and that TaGW2 mediated the ubiquitination and proteasomal degradation of TaARR12.

To study the role of *TaARR12* in regulating drought resistance, the authors generated both overexpression and knockdown lines. Under optimal watering conditions, there were no differences with the wild type; however, after drought stress, the overexpression lines displayed ∼15% plant recovery, the wild type around 48%, and the knockdown lines ∼80%, indicating that *TaARR12* negatively regulates drought resistance. Interestingly, enhanced drought resistance was not linked to yield because there were no differences in kernel development and yield. The authors asked whether the downregulation of *TaARR12* could rescue the severity of drought on the *tagw2* lines. Analysis indicated that the *tagw2 TaARR12* RNAi lines still have a higher kernel size and weight; most importantly, this feature is preserved under water-limited conditions (Figure). The simultaneous depletion of *TaGW2* and *TaARR12* generated higher resistance to drought and higher yield.

**Figure.**
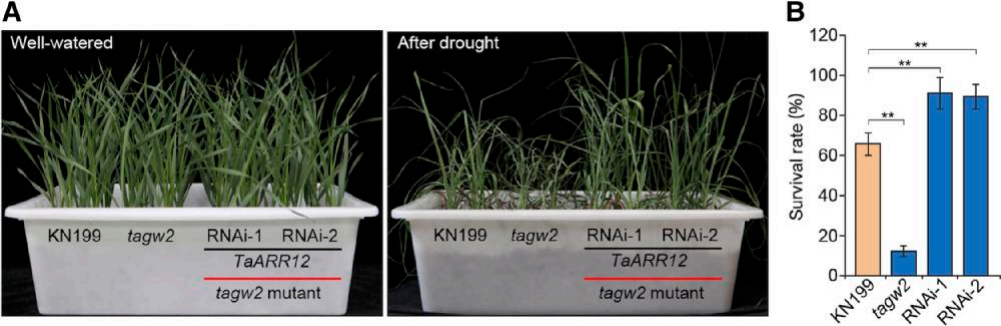
The effects of *TaARR12* and *TaGW2* on wheat drought resistance. **A)** Plant phenotypes of KN199, *tagw2*, and *tagw2 TaARR12* RNAi plants before drought treatment and after rewatering. **B)** Plant survival rates after drought treatment and recovery. Adapted from Li et al. (2023), Figure 5.

The authors went further and explored the genes acting downstream of *TaARR12*. According to luciferase transactivation assays, TaARR12 is a transcriptional repressor. Analysis of the *TaARR12* overexpression transcriptome under well-watered and water-deficit conditions revealed a large set of differentially regulated genes belonging to multiple pathways related to the response to drought. The RNA-seq data combined with DAP-seq (DNA binding sites) allowed the identification of 1,065 upregulated and 1,043 downregulated genes. Most had the cytokinin response motif in their promoters. Finally, the authors selected 2 group-A bZIP genes, potentially regulated by TaARR12 and tested the regulation in vivo. They found that TaARR12 binds the promoter of the *TaABF2* gene on the cytokinin response motif to repress its transcription.

In summary, this work dissected the downstream action of *TaGW2* to uncouple the effect on drought and yield control. The combinatorial action of TaGW2 and TaARR12 is an attractive way to boost drought resistance and yield.
